# PROTOCOL: Discharge programmes for individuals experiencing, or at risk of experiencing, homelessness: A systematic review

**DOI:** 10.1002/cl2.1109

**Published:** 2020-08-24

**Authors:** Jennifer Hanratty, Sarah Miller, Ciara Keenan, John Cowman, Jayne Hamilton, Peter Mackie

**Affiliations:** ^1^ Campbell UK and Ireland, Centre for Evidence and Social Innovation, Queen's University Belfast Belfast UK; ^2^ Department of Social Work Tallaght Adult Mental Health Services Dublin Ireland; ^3^ School of Geography and Planning Cardiff University Cardiff UK

## 1 BACKGROUND

### 1.1 The problem, condition or issue

People who have spent time in an institutional setting, such as prison or in‐patient health services, may be at risk of homelessness upon discharge from the institution (Tsai & Rosenheck, 2015; Winkler et al., 2016). This might be because they were homeless before entering an institutional setting or because previous accommodation arrangements have broken down or are now unsuitable. Those leaving institutional settings are likely to have existing challenges to their health and well‐being and so this population is especially at risk of poor outcomes if discharged into homelessness, unstable housing or accommodation that is no longer suitable for their needs. This review will synthesis the evidence on programmes aimed at preventing or reducing the risk of homelessness for people leaving institutional settings.

#### 1.1.1 Extent of the problem and associated problems

In this review, institutional settings refer to any setting where an individual's accommodation is provided by the institution but provision of accommodation is not the purpose of the institution. Settings can include, but are not limited to, prison, in‐patient treatment (for physical or mental healthcare, addiction treatment), military and young people ageing out of the care system. Those who have been residing in an institutional setting are known to be at higher risk of homelessness upon discharge than the general population (Gulliver, 2015). For example, in the US, between 31% and 46% of youth ageing out of foster care had been homeless at least once by age 26, compared to just 4% of the general population (Dworsky, Napolitano, & Courtney, 2013). A Canadian study of discharge from psychiatric hospital found that 10.5% of people were discharged into homelessness (Forchuk, Russell, Kingston‐MacClure, Turner, & Dill, 2006). Discharge to inappropriate accommodation harms recovery and is a major cause of readmission (Diggle, Butler, Musgrove, & Ward, 2017). Similarly, those discharged from prison are at higher risk of homelessness may have restrictions on where they can and cannot live and face difficulties in accessing accommodation because of their criminal record. In the UK, one‐third of prisoners said they had “no where to go” when leaving prison (Centre for Social Justice, 2010) and both homelessness prior to incarceration and on discharge have been linked to elevated rates of reoffending (Cooper, 2013). Interventions designed to prevent homelessness in this population aim to interrupt this cycle of incarceration, homelessness and reoffending.

Depending on the institutional setting people have been residing in, different groups of people are likely to have different needs. For example, those discharged from in‐patient addiction treatment are likely to need a stable, drug‐free living environment. Whereas youth ageing out of care may need structured practical tapering support to enable them to become self‐sufficient adults. Women may be more likely to also have dependent children and/or need secure accommodation and so housing needs will be different than for those without dependent children. There will also be many individuals with multiple risk factors and complex needs, placing them at even higher risk of homeless and associated negative outcomes. Discharge into shelter accommodation where overcrowding, violence drug use and lack of privacy may be common, is not a suitable place for a person with multiple complex needs. After discharge from institutions, unstable or unsuitable living conditions can contribute to relapse, recidivism, deterioration in health and readmission to hospital (Gulliver, 2015).

#### 1.1.2 The intervention

Discharge programmes involve the coordination and provision of services, including accommodation, for people upon discharge from institutions. These programmes aim to avoid discharging people into homelessness and to reduce the risk of subsequently becoming homeless, with the overarching goal of trying to prevent people entering into a costly cycle of unsafe discharge, readmission, relapse or recidivism (Cooper, 2013; Whiteford & Cornes, 2019). Discharge programmes may be offered to people in a diverse set of circumstances including people; leaving military service; released from prison; being discharged from hospitals, mental health services, addiction treatment or other in‐patient healthcare services; young people ageing out of the care system. Supporting a person to establish suitable stable housing may in turn improve their chances of recovery from illness or addiction, reduce the risk of relapse or recidivism and improve quality of life.

The programmes currently in use in high‐income countries adopt a variety of approaches with different levels of complexity. Programmes primarily seek to address housing needs, either through maintaining previous housing arrangements prior to entry into the institution or to seek new suitable accommodation. Programmes may also offer continued support prior to and following on from discharge, to ensure the persons' housing situation is suitable and sustainable. This could be in the form of paying rent for the individual, facilitating family/partner contact to maintain relationships during time away from home. For example, one simple intervention in a prison context is supporting contact with the family to maintain relationships so the person has a home to return to on release. Other, more complex models, involve the coordination of multiple agencies to enhance the continuity of care and support a person to access services. For example, Critical Time Intervention (Herman et al., 2011) offers care coordination along with direct emotional and practical support over 9 months during the critical discharge period. Another example is a “transition of care” model, where hospital settings work together with community health and social care colleagues, housing organisations and voluntary sector to plan for a person's discharge and effectively communicate with each other to facilitate a smooth transition from the institution to community living with the goal of reducing the need for readmission.

#### 1.1.3 How the intervention might work

Generally, discharge programmes aim to prevent people from being discharged into homelessness or to reduce the risk that they will become homeless due to unsuitable or unsustainable housing. The range of possible approaches is broad but generally, they seek to achieve this aim through assessing individual needs, planning for discharge in advance, establishing communication and coordination between the institution and relevant statutory and voluntary agencies, such as social services, housing agencies, parole office, community health teams to ensure that a person is discharged into suitable accommodation. Some interventions will also provide ongoing support to help each person to access appropriate health and/or social care services to reduce the risk of readmission and support their reintegration into the community. By improving access to suitable accommodation and support services, there is improved opportunity for complete recovery from both physical and mental illness, substance use and reducing the risk of recidivism and improved quality of life.

### 1.2 Why it is important to do this review

There is a significant need to identify and implement effective policies and interventions, and discontinue ineffective practices in order to reduce homelessness. Discharge from institutions is recognised as a major cause of homeless. People who are approaching the transition from an institutional setting may be particularly at risk of homelessness on discharge. To ensure that policymakers avail of the most robust and rigorous evidence to date a systematic review of the literature on interventions aimed at reducing risk and/or incidence of homelessness for this vulnerable population is needed.

This systematic review will be based on evidence already identified in two existing evidence and gap maps (EGMs) commissioned by the Centre for Homelessness Impact (CHI) and built by White, Saran, Teixeira, Fitzpatrick, and Portes (2018). The EGMs present studies on the effectiveness and implementation of interventions aimed at people experiencing, or at risk of experiencing, homelessness. The EGMs were constructed using a comprehensive search strategy including a search of Campbell, PROSPERO and Cochrane databases. The map identified one systematic review relevant to discharge interventions (Kyle & Dunn, 2008). However, this review is focused primarily on people with severe mental illness and is not a review of the effectiveness of discharge programmes. One other possibly overlapping review is by Chambers et al. (2018) on housing interventions for “vulnerable adults”. While there may be some overlap, our review is distinct in its focus on discharge programmes specifically and including any individuals at risk of homelessness and not limited to adults only. Our proposed review is also unique in that we will include evidence on both effectiveness and implementation, including qualitative data, to develop a comprehensive synthesis of which programmes can work, for whom, under what circumstances alongside a synthesis of the common barriers and facilitators for effective implementation.

### 1.3 Objectives

We developed the objective for this review in consultation with the Centre for Homelessness Impact, the team that produced the EGM and consultation with a panel of advisors with expertise and/or experience in the homeless sector. The EGM identified two types of gaps in the evidence base; one where too few primary studies exist and the second where primary studies exist that have not been synthesised. This review is based on a latter gap, the map identified a number of primary studies on the effectiveness of discharge programmes for people transitioning from an institutional setting and no systematic review synthesising that body of evidence. As such, the objective of this review is to synthesise the evidence on the effectiveness of discharge programmes, specifically we aim to answer the following research questions.

1. What is the effect of discharge programmes on outcomes for individuals experiencing or at risk of experiencing homelessness?
2. Do the effect of discharge programmes differ depending on:
  (i) the institutional setting people are discharged from, for example, prison, hospital, substance abuse treatment?
  (ii) complexity of needs?
  (iii) age?
  (iv) the presence of dependent children, in other words, families compared to single individuals?
  (v) sex?
  (vi) race/ethnicity?
3. What implementation and process factors impact intervention delivery (qualitative synthesis)?

## 2 METHODS

### 2.1 Criteria for considering studies for this review

#### 2.1.1 Types of studies

We will include all study designs where a comparison group is used. This includes randomised controlled trials, quasi‐experimental designs, matched comparisons, other study designs that attempt to isolate the impact of the intervention on homelessness using appropriate statistical modelling techniques.

As randomised control trials are accepted as more rigorous than nonrandomised studies, the potential impact of nonrandom study design on effect sizes will be explored as part of the analysis of heterogeneity.

Studies must include an inactive comparison condition that could include the following.

1. No treatment.
2. Treatment as usual where clients receive the normal level of support or intervention. Details of what this consists of will be extracted.
3. Waiting list people are randomly assigned to receive the intervention at a later date. Details of what happens to waitlisted participants will be extracted.
4. Attention control, where participants receive some contact from researchers but both participants and researchers are aware that this is not an active intervention.
5. Placebo where participants perceive that they are receiving an active intervention but the researchers regard the treatment as inactive.

Studies with no control or comparison group, unmatched controls or national comparisons with no attempt to control for relevant covariates will not be included. Case studies, opinion pieces or editorials will not be included.

#### 2.1.2 Types of participants

Persons experiencing, or at risk of experiencing, homelessness residing in an institutional setting, in high‐income countries according to the World Bank country classification. People at risk of homelessness will be defined as those who have a history of homelessness or unstable living arrangements or who has housing prior to admission was or is no longer suitable for their current needs. For example, a person who's prison discharge or parole conditions prevent them from residing with family members.

We will include people of all ages and in any institutional setting including but not limited to military service, social care, in‐patient healthcare, residential treatment for addiction and prison.

#### 2.1.3 Types of intervention

We will include any intervention targeted at people being discharged from any institutional setting, which aims to avoid discharging people from an institutional setting into homelessness or reduce the risk of future homelessness. Interventions primary aim must be to prevent or reduce the risk of future homelessness through planning for suitable stable accommodation prior to discharge. Typically, interventions will involve advance planning prior to discharge and coordination between institutions and housing services. Some interventions will also provide ongoing support to people to enable them to access appropriate health and social care services to support their transition from an institutional setting to community living.

The control or comparison condition can include no services/intervention, services as usual, attention control or waiting list (see study design Section 2.3.3 for more detail).

#### 2.1.4 Types of outcome measures

Given the breath of possible outcomes and measurement tools, we will extract all outcome data relating to seven broad domains. If no useable data are available, we will still include the study in the review but not in the meta‐analysis.

##### Primary outcome domains

1. Housing stability.
2. Health, including substance abuse, mental health, mortality and morbidity.

##### Secondary outcome domains

1. Access to services, including appropriate ongoing community support for individual needs.
2. Crime/criminalisation.
3. Employment and income.
4. Capabilities and well‐being.
5. Cost of intervention.
6. We will also document any unintended adverse events reported.

These domains reflect six out of the seven outcome domains used in the EGM (White, Saran, et al., 2018), with the addition of cost. These outcome domains were developed in consultation with an advisory group of homelessness experts and service providers.

We will also pay particular attention to implementation and acceptability of interventions and will include an analysis of attrition rates or “dropout” from interventions.

#### 2.1.5 Duration of follow‐up

We will include studies with follow‐up of any duration and data relating to all follow‐up points will be extracted. We will conduct separate analysis for each follow‐up period as follows; up to 1 month, 6 months, 1 year, 2 years and more than 2 years postdischarge. The follow‐up analysis will focus on time postdischarge rather than time postintervention as interventions are likely to vary substantially in their duration and because the point of discharge is a crucial transition point.

#### 2.1.6 Types of settings

Relevant institutional settings will include but not be limited to military service, social care, in‐patient healthcare, residential treatment for addiction and prison. Settings to which individuals are discharged may include, but not be limited to, respite care, temporary housing, shelter/hostel, their own home with modifications to make it suitable for current needs, permanent housing.

### 2.2 Search methods for identification of studies

This systematic review will be based on evidence already identified in two EGMs commissioned by the CHI and built by White et al. (2020). The EGMs present studies on the effectiveness and implementation of interventions aimed at people experiencing, or at risk of experiencing, homelessness in high‐income countries. The maps used a comprehensive three‐stage search and mapping process. Stage one was to map the included studies in an existing Campbell review on homelessness (Munthe‐Kass, Berg, & Blaasvær, 2018). Stage two was a comprehensive search of 17 academic databases, three EGM databases and eight systematic review databases for primary studies and systematic reviews. Finally, stage three included web searches for grey literature, scanning reference lists of included studies and consultation with experts to identify additional literature. Sample search terms can be found in the protocol (White et al., 2020). The EGM is maintained and updated periodically and we will report the date of the most recent update of the map in our review.

We will not undertake any additional searching. However, if in the course of contacting authors for additional information or data necessary for conducting analysis and risk or bias assessments, authors provide us with additional eligible studies these would be included.

### 2.3 Data collection and analysis

#### 2.3.1 Description of methods used in primary research

Interventions will include any study measuring the effectiveness of discharge programmes compared to a control group or well‐matched comparison group.

#### 2.3.2 Criteria for determination of independent findings

It is important to ensure that the effects of an individual intervention are only counted once and the following conventions will therefore apply.

Where there are multiple measures reported for the same outcome, we will use robust variance estimation to adjust for effect size dependency (Hedges, Tipton, & Johnson, 2010). The correction for small samples (Tipton & Pustejovsky, 2015) will be implemented when necessary. The exception will be any treatment inherent measures of the outcome of interest, these measurements will be discarded as they risk overestimating the treatment effect.

Where the same outcome construct is measured but across multiple time domains, such as through the collection of both posttest and further follow‐up data, the analysis will be conducted and reported separately for different time points (see above).

Studies comparing multiple treatment and control arms will be discussed with the full author team to decide if eligible intervention arms are similar enough to combine and compare as if they are one intervention group. If not, each intervention arm will contribute separate effect sizes to the meta‐analysis and we will use robust variance estimation to adjust for effect size dependency (Hedges et al., 2010).

In the case of multiple cohorts appearing in one study, we will calculate a simple average, as described above, for the omnibus meta‐analysis. In cases where study authors separate participants into subgroups relating to age, comorbid diagnosis or sex and it's inappropriate to pool their data, these participants will remain independent of each other and will be treated as separate studies which each provide unique information. If different cohorts in a study fall into different subgroups, then they will be considered separately in subgroup analysis but no overall summary of effect will be calculated combining subgroups in those cases. If there are sufficient eligible studies reporting multiple and dependent effect sizes (i.e., occurring in more than 20 eligible studies) then robust variance estimation will be employed. This technique calculates the variance between effect sizes to give the variable of interest a quantifiable standard error. It has been shown to calculate correct results with a minimum of 20–30 individual studies (Hedges et al., 2010) although it performs better with an increased quantity of studies.

#### 2.3.3 Study selection/data extraction

To identify studies from the map that are eligible for inclusion in this review, we will undertake independent double‐blind screening of each title and abstract of all documents in the effectiveness map using EPPI Reviewer 4 software. We will then screen the full text of studies that meet or appear to meet the inclusion criteria, again with independent double‐blind screening. Any disagreements will be resolved in discussion with a third reviewer until we reach a consensus. We will apply the same process to screening documents included in the process evaluation maps to identify studies eligible for inclusion in the qualitative synthesis. We will document the flow of studies through the screening process in a PRISMA flow chart.

The exception to the above will be in the selection of qualitative evidence. Qualitative studies will be selected through purposive sampling. The existing EGM of evidence on implementation has already been coded by the authors of the map. This coding will allow us to easily identify which contain qualitative data on the implementation of discharge programmes. Each potentially relevant study will be reviewed by one author with expertise in qualitative research methods in consultation with the lead author. Studies will be selected through purposive sampling to include studies on discharge interventions, which provide information on implementation of discharge programmes specifically, represent a geographical spread of study locations. Studies will be selected and synthesised until saturation is reached. See section 2.5 on treatment of qualitative studies for more information.

#### 2.3.4 Data extraction and management

Once eligible studies have been found, we will undertake dual data extraction, where two authors will both complete data extraction and risk of bias assessments independently for each study. Coding will be carried out by trained researchers. Any discrepancies in screening or coding will be discussed with the lead author until a consensus is reached.

##### Details of study coding categories

A coding framework has been developed and piloted prior to undertaking data extraction for all included studies using EPPI Reviewer software (Appendix A and B). At a minimum, we will extract the following data: publication details, intervention details including setting, dosage and implementation, delivery personnel, descriptions of the outcomes of interest including instruments used to measure, design and type of trial, sample size of treatment and control groups, data required to calculate Hedges' *g* effect sizes and quality assessment. We will also extract more detailed information on the interventions such as duration and intensity of the programme, timing of delivery, key programme components (as described by study authors) and theory of change. Alongside extracting data on programme components, descriptive information for each of the studies will be extracted and coded to allow for sensitivity and subgroup analysis. This will include information regarding the following.

1. Setting, which type of institutional setting(s) are study participants transitioning from?
2. The study characteristics in relation to design, sample sizes, measures and attrition rates, who funded the study and potential conflict of interests.
3. Demographic variables relating to the participants including age, complexity of needs, dependent children and other relevant population characteristics.

Quantitative data will be extracted to allow for calculation of effect sizes (such as mean change scores and standard error or pre‐ and post‐means and standard deviations or binary 2 × 2 tables). Data will be extracted for the intervention and control group on the relevant outcomes measured in order to assess the intervention effects.

#### 2.3.5 Assessment of risk of bias in included studies

Assessment of methodological quality and potential for bias will be conducted using the second version of the Cochrane Risk of Bias tool for randomised controlled trials (Higgins et al., 2016). Nonrandomised studies will be coded using the ROBINS‐I tool (Sterne et al., 2016). Dual independent screening will be undertaken with any discrepancies discussed and agreed with the lead author. We will include a description of the overall quality of the included studies and graphical representation of study quality using “traffic light tables”, with red indicating low‐quality/high‐risk of bias, yellow for “unclear” and green for high‐quality/low‐risk of bias in the review. We will also integrate consideration of the quality of evidence into any narrative synthesis for each outcome.

#### 2.3.6 Measures of treatment effect

It is anticipated that most outcomes reported will be based upon continuous variables and so the main effect size metric to be used for the purposes of the meta‐analyses will be the standardised mean difference, with its 95% confidence interval. Within this, Hedges' *g* will be used to correct for any small sample bias. Where other effect sizes have been reported, such as Cohen's *d* or risk ratios (for dichotomous outcomes) these will be converted to Hedges' *g* for the purposes of the meta‐analysis using formulae provided in the Cochrane Handbook (Higgins & Green, 2011). Where both are reported, change from baseline metrics will be preferred over endpoint only data. Where both adjusted and unadjusted data are reported, we will also select outcome data that have been adjusted to account for relevant confounding variables over unadjusted data.

#### 2.3.7 Unit of analysis issues

If studies involve group‐level allocation, where possible, data will be included that have been adjusted to account for the effects of clustering, typically through the use of multilevel modelling or adjusting estimates using the intracluster correlation coefficient (ICC). Where the effects of clustering have not been taken into account, estimates of effect size will be adjusted following guidance in the Cochrane Handbook. If ICC is not reported, external estimates will be obtained from studies that provide the best match on outcome measures and types of clusters from existing databases of ICCs (Ukoumunne, Gulliford, Chinn, Sterne, & Burney, 1999) or other similar studies within the review. If a crossover trial is included in the review, we will only use data reported for the first part of the trial before the crossover point.

#### 2.3.8 Dealing with missing data

If study reports do not contain sufficient data to allow calculation of effect size estimates, authors will be contacted to obtain necessary summary data, such as means and standard deviations or standard errors. If no information is forthcoming, the study cannot be included in meta‐analysis and will instead be included in a narrative synthesis. If data are missing due to drop out from the study, we will use data where missing data have been imputed, where reported. If not reported, we will include the data but consider the effect of inclusion of studies with more than 20% attrition in sensitivity analysis.

#### 2.3.9 Assessment of heterogeneity

Heterogeneity will be assessed first, through visual inspection of the forest plot and checking for overlap of confidence intervals and second through the *Q*, *I* ^2^ and Tau^2^ statistics. Sources of heterogeneity that we anticipate are differences in the intervention logic and the population, specifically populations with varying levels of pre‐existing risk for negative outcomes of interest.

#### 2.3.10 Assessment of reporting biases

A funnel plot and Egger's linear regression test will be included to check for publication bias across included studies (Sterne & Egger, 2005). Where the funnel plot is asymmetrical, this indicates either publication bias or bias which relates to smaller studies showing larger treatment effects. The trim and fill method will be used where the funnel plot is asymmetrical (Higgins & Green, 2011), this is a nonparametric technique that removes the smaller studies causing irregularity until there is a new symmetrical pooled estimate, the studies that were eliminated were then filled back in to reflect the new estimate. We acknowledge that tests for funnel plot asymmetry are limited by low power, are not appropriate for use with fewer than 10 studies, should not be used when study effect sizes are similar and that results of the statistical tests should not be used alone without visual inspection of the funnel plot and interpreted with caution.

### 2.4 Data synthesis

#### 2.4.1 Approach to meta‐analysis

Given the diverse range of interventions that this review is likely to find, random effects models, using inverse‐variance estimation, will be used as the basis for pairwise meta‐analysis. The analysis will be conducted using *R* and the range of commands externally developed to conduct meta‐analysis with *R* such as metafor.

##### Main effects (objective 1)

The main effects analysis, synthesising the evidence in relation to the effects of discharge programmes in general, will be undertaken using the approach to meta‐analysis outlined above for each primary and secondary outcome in turn, with separate analysis for follow‐up of different duration (see section 2.1.5 duration of follow‐up). We will conduct meta‐analysis if at least two studies have reported the same outcome either using the same measurement tool or a tool that is sufficiently similar to reasonably assume that the effect measured reflects the same underlying concept. If studies do not measure or report outcomes using measurement tools that are similar enough to be sensibly combined in meta‐analysis, studies will instead be narratively synthesised.

##### Sensitivity analysis

For each outcome, the following sensitivity analyses will also be undertaken to assess whether there are potential influences relating to the following.

1. Studies that appear to exert an undue influence on findings.
2. Study quality (studies with a “high” or “unclear” risk of bias on 3 or more of the 7 risk of bias domains in the Cochrane Risk of Bias assessment will be coded as low quality).
3. The inclusion of studies with more than 20% attrition.

##### Subgroup analysis and investigation of heterogeneity

Assessment of differential effectiveness in relation to age, complexity of needs, family (dependent children) or single, institutional setting or other subgroups/populations identified in included studies (objective 2).

Eligible studies will be coded in terms of the following.

1. The institutional setting people have been residing in.
2. Age (under 25 or over 25).
3. Complexity of needs, this will be defined based on mental health, physical health, substance use/abuse status and any other relevant factors).
4. Dependent children (comparing interventions for families including dependent children and individuals without dependent children).
5. Sex.
6. Race/ethnicity.
7. Duration of intervention.

Subgroup analyses will then be conducted in relation to each of the factors above (institution, age, complexity of needs, dependent children, race/ethnicity and sex) for each of the primary and secondary outcomes. The subgroup analyses (based upon random‐effect models) will group studies by subcategory and estimate overall effects sizes for each. Subgroup analyses will only be carried out where studies included in the subgroup analysis are sufficiently similar to each other in all other respects, such as whether the interventions delivered to younger and older people are similar enough to be confident that the subgroup analysis reflects differences in the effectiveness for different populations rather than different intervention effects.

### 2.5 Treatment of qualitative research

The qualitative research that will be included in this review is based upon existing evidence collated through an EGM constructed by White, Saran, et al. (2018) and White, Wood, and Fitzpatrick (2018). The EGM was commissioned by the CHI and presents 292 qualitative process evaluations on the implementation issues of interventions designed to target homelessness. These were downloaded from EPPI reviewer on May 10, 2019. These qualitative studies will be screened for relevance to the current review.

The EGM categorises the included studies into broad categories of barriers and facilitators to the implementation of interventions. These categories were developed using an iterative process and were initially based on the implementation science framework (Aarons, Hurlburt, & Horwitz, 2011). The categories were then independently piloted against process evaluations and agreement was reached by researchers in the Campbell Collaboration, Campbell UK and Ireland, and Herriot‐Watt University. The five broad categories agreed are contextual factors, policymakers/funders, programme managers/implementing agency, staff/case workers and recipients. The review team recognise that in the majority of discharge interventions, more than one of the agreed categories could act as a factor that impacts positively or negatively on the effectiveness of the intervention, or both in some cases. This potential overlap reflects the complexity of the implementation of the interventions and the multifaceted evaluation tools needed within this review. For this reason, the review team have decided to focus on factors that influence the implementation of discharge programmes in order to formulate a coherent thematic synthesis.

We will include process evaluations and other relevant qualitative studies that provide data that enables a deeper understanding of why the discharge programmes included in the quantitative synthesis do (or do not) work as intended, for whom and under what circumstances. We will conduct a thematic synthesis (Thomas & Harden, 2008) to generate new themes and create meaningful relationships between these themes (Flemming, Booth, Garside, Tunçalp, & Noyes, 2019).

The quality of these mixed methods studies will be assessed using a tool developed by White and Keenan (2018). The tool is similar to the fidelity assessment used by Stergiopoulos, Hwang, O'Campo, Jeyaratnam, and Kruk (2013) and aims to provide an accurate account of the eligible qualitative studies. The tool will consider methodology, recruitment and sampling, bias, ethics, analysis and findings. We will also describe the characteristics of included qualitative studies in terms of what qualitative methods have been used to capture this rich data, the number of interviews/focus groups/observations that have taken place, who participated and the nature of qualitative data collection (type and time taken).

The implementation and process evaluations will be critical in this analysis, and data gathered from observations, focus groups and interviews will add an essential and unique human perspective to this review. By including an element of qualitative evidence synthesis in our review, we hope to provide a more robust and rich review of the evidence base.

## AUTHOR CONTRIBUTIONS

P. M., J. C., J. Han. and C. K. prepared the content. J. Han., S. M. and C. K. contributed to systematic review methods and statistical analysis. J. Han. and J. Ham. contributed to qualitative evidence synthesis. The review will be undertaken by systematic review specialists within the Campbell UK and Ireland Centre. S. M. will be the Principal Investigator (PI) of the project and will have overall responsibility for its conduct and delivery. J. H. will be responsible for the day‐to‐day operation of the reviews. This review will be supported by specialist input from C. K. alongside research support from two full‐time research assistants (Hamilton and Coughlan). J. H. has worked in evidence synthesis since 2012 and published reviews with Campbell, Cochrane and NIHR Health Technology Assessment amongst others. She is an Editor with Campbell Education Coordinating group, on the editorial board of the Campbell Knowledge Translation and Implementation Group, and represents Campbell UK and Ireland on the advisory board for Evidence Synthesis Ireland. S. M. is Director of Campbell UK and Ireland. She is co‐chair and co‐editor of the Campbell Education Coordinating Group and also Deputy Director of the Centre for Evidence and Social Innovation, within which she leads the What Works in Schools programme of research. She has considerable methodological and statistical expertise, which includes the conduct and analysis of randomised controlled trials as well as systematic reviews and meta‐analyses. C. K. has acquired 6‐year experience working across 15 Systematic Reviews. She is currently co‐convenor of the Campbell Collaboration's Information Scientist Network; methods editor for the Campbell Collaboration's Education coordinating group; and founder and editor of the meta‐evidence blog. J. C. is a Housing Coordinator in mental health services in Dublin. He is a qualified social worker who had worked in specialist housing roles since 2013. His main focus has been on promoting recovery‐oriented housing and supports for people with mental health disabilities, in particular, ways to elicit and incorporate the service user's subjective needs and preferences. He has developed several innovative interagency housing projects and also been involved in research and evaluation. He has completed several housing‐related research studies, one of which led to the MSc in Mental Health in Trinity College Dublin (2008). In addition to his main role, he is currently a PhD student at Queen's University Belfast. His PhD study is exploring the housing needs of people in psychiatric in‐patient care and how those needs can be most effectively met. J. Ham. is a qualified primary school teacher and has been involved in qualitative research methods and data collection across her own undergraduate and postgraduate studies and has recently attended training qualitative evidence synthesis methods. P. M. is a Reader at Cardiff University. The primary focus of Peter's research and advisory work is on homelessness policy and legislation, particularly in relation to homelessness prevention. Peter is a research advisor to the European Umbrella body for homelessness organisations (FEANTSA) and the chair of the Wales Housing Research Network.

## APPENDIX A DATA COLLECTION FORM FOR HOMELESSNESS REVIEWS

**Table:**

1. Bibliographic information	
Article ID	FREE TEXT
Linked articles	FREE TEXT
Extracted by	FREE TEXT
Checked by	FREE TEXT
Year of publication	FREE TEXT
Type of publication	1. Journal article 2. Book/book chapter 3. Government report 4. Conference proceedings 5. Presentation 6. Thesis or dissertation 7. Unpublished report 8. Other (please specify)
Location of study	1. UK 2. ROI 3. Rest of Europe 4. USA 5. Canada 6. South America 7. Central America 8. Oceania 9. Middle‐East 10. Asia 11. Africa 12. Other (please specify)
*The location in which the study is set **not** where the study authors are based*.	
	Not specified
Study funding sources	1. Research council funding 2. University scholarships and bursaries 3. Salaried research assistantships from university departments 4. Grants or loans from trusts and charities 5. Local enterprise initiatives 6. Company sponsorship 7. Government loans 8. EU scholarships 9. Industry sponsorship 10. Other (please specify)
Possible conflicts of interest	1. Yes, possible/definite conflict of interest 2. No, study appears to be free of CoI 3. Can't tell

2. Participant information	
Recruitment setting	1. Clinical setting 2. Accommodation for individuals experiencing homelessness 3. Family home 4. The street 5. Community setting 6. Referred by friends or family 7. Referred by medical health professional 8. Housing agency 9. Other (please specify)
*Where were participants recruited from?*	
Homelessness Status at intake	1. Sleeping “Rough” (or rooflessness) 2. Temporary accommodation 3. Insecure accommodation 4. Inadequate Accommodation 5. Involuntary sharing, for example, domestic violence 6. Hidden/concealed homelessness 7. Other (please specify)
*Describe the housing status of the sample at intake and/or any information given about housing status prior to intake. Tick all that apply and try to extract numbers were available*.	
*Homelessness is defined as those individuals who are sleeping* “*rough*” *(sometimes defined as street homeless), those in temporary accommodation (such as shelters and hostels), those in insecure accommodation (such as those facing eviction or in abusive or unsafe environments), and those in inadequate accommodation (environments which are unhygienic and/or overcrowded)*.	
	Not specified
Geographical context	1. Urban 2. Rural 3. Suburban 4. Mixed 5. Other (please specify)
*Where participants receive treatment?*	
	Not specified
Sex	FREE TEXT
*% (Actual number)*	
Age	1. Under 25 2. 25 and over
*Extract mean age, SD and range*	
*Choose multiple options if the analysis is reported separately for different age groups*	
Complexity of needs	1. Poor physical health 2. Poor mental health 3. Incarceration 4. Substance abuse issues 5. Care leaver 6. Limited access to integrated support services 7. High risk of harm and/or exploitation 8. Other (please specify)
*What other challenges does the individual face, if any, aside from the risk or experience of homelessness?*	
*High risk of harm and/or exploitation—for example, women in shelters, newcomer families, refugee/asylum seeker, care leavers*	
	Not Relevant
	Not Specified
Mental health status	1. Receiving treatment 2. Not receiving treatment 3. Other (please specify)
	Not relevant
	Not Specified
Substance use status	1. Receiving treatment 2. Not receiving treatment 3. Other (please specify)
Not relevant	
Not specified	
Homelessness status	1. Sleeping “rough” 2. Temporary accommodation 3. Insecure accommodation 4. Inadequate accommodation 5. Other (please specify)
*Homelessness is defined as those individuals who are sleeping “rough” (sometimes defined as street homeless), those in temporary accommodation (such as shelters and hostels), those in insecure accommodation (such as those facing eviction or in abusive or unsafe environments), and those in inadequate accommodation (environments which are unhygienic and/or overcrowded)*	
	Not Specified
Family versus no family	1. Family 2. Nonfamily
*Family* = *any child involved*	
	Not specified
*Nonfamily* = *single person or couple without children*	
*If mixed sample select both and describe*	
Sample size of treatment group	FREE TEXT
*Number of people assigned to treatment. If more than one treatment group extract all and be clear which group is which*.	
Sample size of control group	FREE TEXT
*Number of people assigned to control. If more than one control group extract all and be clear which group is which*.	

3. Intervention information	
How many intervention arms in this trial?	FREE TEXT
*List how many study arms there are and given each a name, for example, Intervention* = *Critical Time Intervention; Control* = *Treatment as usual*	
*If there is more than one intervention arm go to the "Study Arm" tab and add the RELEVANT study arms. You must then extract data for each relevant study arm*	
Name of intervention	FREE TEXT
*Write in the name of the program, intervention, or treatment under study. This may be specific like “critical time intervention” or it may be something more generic like “supported housing”*	
Briefly Describe the intervention	FREE TEXT
*Briefly describe the intervention, what participants are offered and any important factors such as conditionality, nature of housing, case management, substance abuse treatment included etc*	
Theory of change	FREE TEXT
*How does the intervention aim to bring about change? What is the underlying theoretical rationale for why the intervention might work to improve outcomes?*	
*If not specified write "not specified"*	
What is the size of accommodation/How many beds?	FREE TEXT
Duration of treatment period from start to finish	FREE TEXT
*In the dosage items, we are interested in the amount of treatment received by the participants. If the treatment was delivered directly to participants, the authors will probably provide at least some information about dosage and you can code these items accordingly. If minimal information is provided, you should try to give estimates for these items if you can come up with a reasonable estimate*.	
Timing	1. Once a month 2. Less than weekly 3. Once a week 4. 1‐2 times a week 5. times a week 6. 2‐3 times a week 7. times a week 8. 3‐4 times a week 9. times a week 10. Daily contact
*Frequency of contact between participants and provider/program activity*	
	Can't estimate
Length of each individual session	FREE TEXT
*How long does each contact/session last?*	
Study personnel	1. Graduate Researcher 2. Grad/Undergrad Students 3. Author 4. Homelessness professional
*The primary individual/s who have direct contact with the participants served by the program*.	
*If the report is the author's dissertation (or based on the author's dissertation), then code as "Graduate Researcher"*	*Includes case manager, social worker, outreach worker*
*If the delivery is performed by graduate or undergraduate students assisting the author then select "Grad/Undergrad Students"*	5. Peers 6. Interventionist (not hired by researcher) 7. Interventionist (hired by researcher) 8. Self‐directed 9. Medical professionals 10. Other (please specify)
*Code “Self‐directed” for studies where electronic/computer programs are used*.	
*If the intervention is solely environmental i.e. community housing, then code “environmental change”*	
	Not Specified
Did provider receive specialised training?	1. Yes 2. The interventionist IS program developer 3. No
*This refers to whether or not the “interventionist” received specialised training to equip them to deliver the intervention proficiently*.	
	Not specified
Resource requirements	FREE TEXT
*Time, staff, housing provision etc*	
Cost	FREE TEXT

4a. Study design	
Design	1. Randomised control trial
*The studies included in all reviews must include an intervention group and at least one untrained control group. Control groups can include placebo, no treatment, waitlist, or treatments vs “treatment as usual”. Any study which includes one group pre‐test/post‐test or in which a treatment group is only compared to another treatment group will not be eligible for inclusion*.	*Individual or cluster randomised*
	2. Nonrandomised control trial
What do control subjects receive?	1. Placebo 2. Treatment as usual 3. No treatment
1. *Placebo (or attention) treatment. Group gets some attention or a sham treatment* 2. *Treatment as usual. Group gets “usual” handling instead of some special treatment*. 3. *No treatment. Group gets no treatment at all*.	
	Not specified
Unit of allocation	1. Individual 2. Group 3. Regions 4. Other (please specify)
*Individual (i.e., some were assigned to treatment group, some to comparison group)*	
*Group (i.e., whole subsets assigned to treatment and comparison groups)*	
	Not specified
*Regions (i.e., region assigned as an intact unit)*	
Method of assignment	1. Randomly after matching 2. Randomly without matching 3. Regression discontinuity design 4. Cluster assigned 5. Wait list control 6. Nonrandom, but matched 7. Other (please specify)
*Method of group assignment. How participants/units were assigned to groups. This item focuses on the initial method of assignment to groups, regardless of subsequent degradations due to attrition, refusal, etc. prior to treatment onset*.	
1. *Randomly after matching, yoking, stratification, blocking, etc. The entire sample is matched or blocked first, then assigned to treatment and comparison groups within pairs or blocks. This does not refer to blocking after treatment for the data analysis*. 2. *Randomly without matching, etc. This also includes cases when every other person goes to the control group*. 3. *Regression discontinuity design: quantitative cutting point defines groups on some continuum (this is rare)*. 4. *Cluster assigned, this is to be used in cluster assignment studies only, specify the number of clusters in the treatment group and the number of clusters in control*. 5. *Wait list control or other quasi‐random procedure presumed to produce comparable groups (no obvious differences). This applies to groups which have individuals apparently randomly assigned by some naturally occurring process, for example, first person to walk in the door. The key here is that the procedure used to select groups doesn't involve individual characteristics of persons so that the groups generated should be essentially equivalent*. 6. *Non‐random, but matched: Matching refers to the process by which comparison groups are generated by identifying individuals or groups that are comparable to the treatment group using various characteristics of the treatment group. Matching can be done individually, for example, by selecting a control subject for each intervention subject who is the same age, sex, and so forth, or on a group basis*.	
	Not specified
Was there >20% attrition in either/both groups?	FREE TEXT
*Attrition occurs when participants are lost from an intervention over time or over a series of sequential processes. Studies may describe this as “lost to follow‐up”, or “drop outs”*	

4b. Nonrandom studies	
How were groups matched?	1. Matched on pretest measure 2. Matched on personal characteristics 3. Matched on demographics 4. Groups weren't matched 5. Other (please specify)
*If matching was used prior to assignment of condition, how were groups matched?*	
	Not specified
Was the equivalence of groups tested at pre‐test?	FREE TEXT
Results of statistical comparisons of pre‐test differences	1. No statistically significant differences 2. Significant differences judged unimportant by coder 3. Significant differences judged of uncertain importance by coder 4. Significant differences judged important by coder 5. Other (please specify)
Were there pretest adjustments?	FREE TEXT

5. Qualitative information	
Qualitative methods used	FREE TEXT
Data analysis technique and procedure	FREE TEXT
Was the intervention implemented as intended?	1. Yes 2. No
	Not specified
How was this measured?	FREE TEXT
What implementation and process factors impact intervention delivery?	1. Contextual factors 2. Policymakers/funders 3. Programme managers/Implementing agency, 4. Staff/case workers 5. Recipients

6. Assessing quality in RCTs (Cochranes ROB2 tool)	
Domain 1: Risk of bias arising from the randomisation process	
1.1 Was the allocation sequence random?	1. Yes 2. Probably yes 3. Probably no 4. No
1.2 Was the allocation sequence concealed until participants were enrolled and assigned to interventions?	1. Yes 2. Probably yes 3. Probably no 4. No
1.3 Did baseline differences between intervention groups suggest a problem with the randomisation process?	1. Yes 2. Probably yes 3. Probably no 4. No
Risk‐of‐bias judgement	1. Low 2. High 3. Some concerns
Optional: What is the predicted direction of bias arising from the randomisation process?	1. Favours experimental 2. Favours comparator 3. Towards null 4. Away from null 5. Unpredictable
Domain 2: Risk of bias due to deviations from the intended interventions (effect of assignment to intervention)	
2.1. Were participants aware of their assigned intervention during the trial?	1. Yes 2. Probably yes 3. Probably no 4. No
2.2. Were carers and people delivering the interventions aware of participants' assigned intervention during the trial?	1. Yes 2. Probably yes 3. Probably no 4. No
2.3. If Y/PY/NI to 2.1 or 2.2: Were there deviations from the intended intervention that arose because of the experimental context?	1. Yes 2. Probably yes 3. Probably no 4. No
2.4. If Y/PY to 2.3: Were these deviations from intended intervention balanced between groups?	1. Yes 2. Probably yes 3. Probably no 4. No
2.5 If N/PN/NI to 2.4: Were these deviations likely to have affected the outcome?	1. Yes 2. Probably yes 3. Probably no 4. No
2.6 Was an appropriate analysis used to estimate the effect of assignment to intervention?	1. Yes 2. Probably yes 3. Probably no 4. No
2.7 If N/PN/NI to 2.6: Was there potential for a substantial impact (on the result) of the failure to analyse participants in the group to which they were randomised?	1. Yes 2. Probably yes 3. Probably no 4. No
Risk‐of‐bias judgement	1. Low 2. High 3. Some concerns
Optional: What is the predicted direction of bias due to deviations from intended interventions?	1. Favours experimental 2. Favours comparator 3. Towards null 4. Away from null 5. Unpredictable
Domain 3: Missing outcome data	
3.1 Were data for this outcome available for all, or nearly all, participants randomised?	1. Yes 2. Probably yes 3. Probably no 4. No
3.2 If N/PN/NI to 3.1: Is there evidence that result was not biased by missing outcome data?	1. Yes 2. Probably yes 3. Probably no 4. No
3.3 If N/PN to 3.2: Could missingness in the outcome depend on its true value?	1. Yes 2. Probably yes 3. Probably no 4. No
3.4 If Y/PY/NI to 3.3: Do the proportions of missing outcome data differ between intervention groups?	1. Yes 2. Probably yes 3. Probably no 4. No
3.5 If Y/PY/NI to 3.3: Is it likely that missingness in the outcome depended on its true value?	1. Yes 2. Probably yes 3. Probably no 4. No
Risk‐of‐bias judgement	1. Low 2. High 3. Some concerns
Optional: What is the predicted direction of bias due to missing outcome data?	1. Favours experimental 2. Favours comparator 3. Towards null 4. Away from null 5. Unpredictable
Domain 4: Risk of bias in measurement of the outcome	
4.1 Was the method of measuring the outcome inappropriate?	1. Yes 2. Probably yes 3. Probably no 4. No
4.2 Could measurement or ascertainment of the outcome have differed between intervention groups?	1. Yes 2. Probably yes 3. Probably no 4. No
4.3 If N/PN/NI to 4.1 and 4.2: Were outcome assessors aware of the intervention received by study participants?	1. Yes 2. Probably yes 3. Probably no 4. No
4.4 If Y/PY/NI to 4.3: Could assessment of the outcome have been influenced by knowledge of intervention received?	1. Yes 2. Probably yes 3. Probably no 4. No
4.5 If Y/PY/NI to 4.4: Is it likely that assessment of the outcome was influenced by knowledge of intervention received?	1. Yes 2. Probably yes 3. Probably no 4. No
Risk‐of‐bias judgement	1. Low 2. High 3. Some concerns
Optional: What is the predicted direction of bias in measurement of the outcome?	1. Favours experimental 2. Favours comparator 3. Towards null 4. Away from null 5. Unpredictable
Domain 5: Risk of bias in selection of the reported result	
5.1 Was the trial analysed in accordance with a prespecified plan that was finalised before unblinded outcome data were available for analysis?	1. Yes 2. Probably yes 3. Probably no 4. No
Is the numerical result being assessed likely to have been selected, on the basis of the results, from:	
5.2. multiple outcome measurements (e.g., scales, definitions, time points) within the outcome domain?	1. Yes 2. Probably yes 3. Probably no 4. No
5.3 multiple analyses of the data?	1. Yes 2. Probably yes 3. Probably no 4. No
Risk‐of‐bias judgement	1. Low 2. High 3. Some concerns
Optional: What is the predicted direction of bias due to selection of the reported result?	1. Favours experimental 2. Favours comparator 3. Towards null 4. Away from null 5. Unpredictable
Overall risk of bias	
Risk‐of‐bias judgement	1. Low 2. High 3. Some concerns

7. Assessing quality in nonrandom control trials (ROBINS‐I tool)	
Bias due to confounding	
1.1 Is there potential for confounding of the effect of intervention in this study?	1. Yes 2. Probably yes 3. Probably no 4. No
If N/PN to 1.1: the study can be considered to be at low risk of bias due to confounding and no further signalling questions need be considered	
If Y/PY to 1.1: determine whether there is a need to assess time‐varying confounding:	1. Yes 2. Probably yes 3. Probably no 4. No
1.2. Was the analysis based on splitting participants’ follow up time according to intervention received?	1. Yes 2. Probably yes 3. Probably no 4. No
If N/PN, answer questions relating to baseline confounding (1.4 to 1.6)	
If Y/PY, go to question 1.3.	
1.3. Were intervention discontinuations or switches likely to be related to factors that are prognostic for the outcome?	1. Yes 2. Probably yes 3. Probably no 4. No
If N/PN, answer questions relating to baseline confounding (1.4 to 1.6)	
If Y/PY, answer questions relating to both baseline and time‐varying confounding (1.7 and 1.8)	
Questions relating to baseline confounding only	
1.4. Did the authors use an appropriate analysis method that controlled for all the important confounding domains?	1. Yes 2. Probably yes 3. Probably no 4. No
1.5. If Y/PY to 1.4: Were confounding domains that were controlled for measured validly and reliably by the variables available in this study?	1. Yes 2. Probably yes 3. Probably no 4. No
1.6. Did the authors control for any post‐intervention variables that could have been affected by the intervention?	1. Yes 2. Probably yes 3. Probably no 4. No
Questions relating to baseline and time‐varying confounding	
1.7. Did the authors use an appropriate analysis method that controlled for all the important confounding domains and for time‐varying confounding?	1. Yes 2. Probably yes 3. Probably no 4. No
1.8. If Y/PY to 1.7: Were confounding domains that were controlled for measured validly and reliably by the variables available in this study?	1. Yes 2. Probably yes 3. Probably no 4. No
Risk‐of‐bias judgement	1. Low 2. Moderate 3. Serious 4. Critical
Optional: What is the predicted direction of bias due to confounding?	1. Favours experimental 2. Favours comparator 3. Unpredictable
Bias in selection of participants into the study	
2.1. Was selection of participants into the study (or into the analysis) based on participant characteristics observed after the start of intervention?	1. Yes 2. Probably yes 3. Probably no 4. No
If N/PN to 2.1: go to 2.4	
2.2. If Y/PY to 2.1: Were the post‐intervention variables that influenced selection likely to be associated with intervention?	1. Yes 2. Probably yes 3. Probably no 4. No
2.3 If Y/PY to 2.2: Were the post‐intervention variables that influenced selection likely to be influenced by the outcome or a cause of the outcome?	1. Yes 2. Probably yes 3. Probably no 4. No
2.4. Do start of follow‐up and start of intervention coincide for most participants?	1. Yes 2. Probably yes 3. Probably no 4. No
2.5. If Y/PY to 2.2 and 2.3, or N/PN to 2.4: Were adjustment techniques used that are likely to correct for the presence of selection biases?	1. Yes 2. Probably yes 3. Probably no 4. No
Risk‐of‐bias judgement	1. Low 2. Moderate 3. Serious 4. Critical
Optional: What is the predicted direction of bias due to selection of participants into the study?	1. Favours experimental 2. Favours comparator 3. Towards null 4. Away from null 5. Unpredictable
Bias in classification of interventions	
3.1 Were intervention groups clearly defined?	1. Yes 2. Probably yes 3. Probably no 4. No
3.2 Was the information used to define intervention groups recorded at the start of the intervention?	1. Yes 2. Probably yes 3. Probably no 4. No
3.3 Could classification of intervention status have been affected by knowledge of the outcome or risk of the outcome?	1. Yes 2. Probably yes 3. Probably no 4. No
Risk‐of‐bias judgement	1. Low 2. Moderate 3. Serious 4. Critical
Optional: What is the predicted direction of bias due to classification of interventions?	1. Favours experimental 2. Favours comparator 3. Towards null 4. Away from null 5. Unpredictable
Bias due to deviations from intended interventions	
If your aim for this study is to assess the effect of assignment to intervention, answer questions 4.1 and 4.2	
4.1. Were there deviations from the intended intervention beyond what would be expected in usual practice?	1. Yes 2. Probably yes 3. Probably no 4. No
4.2. If Y/PY to 4.1: Were these deviations from intended intervention unbalanced between groups *and* likely to have affected the outcome?	1. Yes 2. Probably yes 3. Probably no 4. No
If your aim for this study is to assess the effect of starting and adhering to intervention, answer questions 4.3 to 4.6	
4.3. Were important co‐interventions balanced across intervention groups?	1. Yes 2. Probably yes 3. Probably no 4. No
4.4. Was the intervention implemented successfully for most participants?	1. Yes 2. Probably yes 3. Probably no 4. No
4.5. Did study participants adhere to the assigned intervention regimen?	1. Yes 2. Probably yes 3. Probably no 4. No
4.6. If N/PN to 4.3, 4.4 or 4.5: Was an appropriate analysis used to estimate the effect of starting and adhering to the intervention?	1. Yes 2. Probably yes 3. Probably no 4. No
Risk‐of‐bias judgement	1. Low 2. Moderate 3. Serious 4. Critical
Optional: What is the predicted direction of bias due to deviations from the intended interventions?	1. Favours experimental 2. Favours comparator 3. Towards null 4. Away from null 5. Unpredictable
Bias due to missing data	
5.1 Were outcome data available for all, or nearly all, participants?	1. Yes 2. Probably yes 3. Probably no 4. No
5.2 Were participants excluded due to missing data on intervention status?	1. Yes 2. Probably yes 3. Probably no 4. No
5.3 Were participants excluded due to missing data on other variables needed for the analysis?	1. Yes 2. Probably yes 3. Probably no 4. No
5.4 If PN/N to 5.1, or Y/PY to 5.2 or 5.3: Are the proportion of participants and reasons for missing data similar across interventions?	1. Yes 2. Probably yes 3. Probably no 4. No
5.5 If PN/N to 5.1, or Y/PY to 5.2 or 5.3: Is there evidence that results were robust to the presence of missing data?	1. Yes 2. Probably yes 3. Probably no 4. No
Risk of bias judgement	1. Low 2. Moderate 3. Serious 4. Critical
Optional: What is the predicted direction of bias due to missing data?	1. Favours experimental 2. Favours comparator 3. Towards null 4. Away from null 5. Unpredictable
Bias in measurement of outcomes	
6.1 Could the outcome measure have been influenced by knowledge of the intervention received?	1. Yes 2. Probably yes 3. Probably no 4. No
6.2 Were outcome assessors aware of the intervention received by study participants?	1. Yes 2. Probably yes 3. Probably no 4. No
6.3 Were the methods of outcome assessment comparable across intervention groups?	1. Yes 2. Probably yes 3. Probably no 4. No
6.4 Were any systematic errors in measurement of the outcome related to intervention received?	1. Yes 2. Probably yes 3. Probably no 4. No
Risk of bias judgement	1. Low 2. Moderate 3. Serious 4. Critical
Optional: What is the predicted direction of bias due to measurement of outcomes?	1. Favours experimental 2. Favours comparator 3. Towards null 4. Away from null 5. Unpredictable
Bias in selection of the reported result	
Is the reported effect estimate likely to be selected, on the basis of the results, from:	
7.1 multiple outcome measurements within the outcome domain?	1. Yes 2. Probably yes 3. Probably no 4. No
7.2 multiple analyses of the intervention‐outcome relationship?	1. Yes 2. Probably yes 3. Probably no 4. No
7.3 different subgroups?	1. Yes 2. Probably yes 3. Probably no 4. No
Risk of bias judgement	1. Low 2. Moderate 3. Serious 4. Critical
Optional: What is the predicted direction of bias due to selection of the reported result?	1. Favours experimental 2. Favours comparator 3. Towards null 4. Away from null 5. Unpredictable
Overall risk of bias	
Risk‐of‐bias judgement	1. Low 2. Moderate 3. Serious 4. Critical

8. Assessing quality in qualitative studies (White and Keenan tool)	
Are the evaluation questions clearly stated?	1. Yes 2. No
Is the qualitative methodology described?	1. Yes 2. No
Is the qualitative methodology appropriate to address the evaluation questions?	1. Yes 2. No 3. Insufficient detail
Is the recruitment or sampling strategy described?	1. Yes 2. No
Is the recruitment or sampling strategy appropriate to address the evaluation questions?	1. Yes 2. No 3. Insufficient detail
Are the researcher's own position, assumptions and possible biases outlined?	1. Yes 2. No
Have ethical considerations been sufficiently considered?	1. Yes 2. No 3. Insufficient detail
Is the data analysis approach adequately described?	1. Yes 2. No
Is the data analysis sufficiently rigorous?	1. Yes 2. No
Is there a clear statement of findings?	1. Yes 2. No
Are the research findings useful?	1. Yes 2. No
